# Comparison of systemic immunoinflammatory biomarkers for assessing severe abdominal aortic calcification among US adults aged≥40 years: A cross-sectional analysis from NHANES

**DOI:** 10.1371/journal.pone.0325949

**Published:** 2025-06-24

**Authors:** Quankai Cheng, Chang Liu, Haicheng Zhong, Ziming Wang, Sheng Zhou, Jingjing Sun, Sihai Zhao, Jie Deng

**Affiliations:** 1 Department of Cardiology, The Second Affiliated Hospital of Xi’an Jiaotong University, Xi’an, Shaanxi, China; 2 Department of Respiratory and Critical Care Medicine, The Second Affiliated Hospital of Xi’an Jiaotong University, Xi’an, Shaanxi, China; 3 Institute of Cardiovascular Science, Translational Medicine Institute, Xi’an Jiaotong University Health Science Center, Xi’an, Shaanxi, China.; University of Montenegro-Faculty of Medicine, MONTENEGRO

## Abstract

**Objective:**

Several novel biomarkers, including the systemic immune-inflammation index (SII), systemic inflammation response index (SIRI), aggregate index of systemic inflammation (AISI), platelet-lymphocyte ratio (PLR), neutrophil-lymphocyte ratio (NLR), and monocyte-lymphocyte ratio (MLR), are linked to the systemic immunity inflammation response and the odds and severity of abdominal aortic calcification (AAC). However, still no previous research has systematically compared their association with severe AAC.

**Methods:**

This study utilized a cross-sectional approach, examining a cohort of 3,047 adults from National Health and Nutrition Examination Survey (NHANES). Weighted logistic regression was utilized to investigate the associations between a range of immunoinflammatory biomarkers and the likelihood of severe AAC. Segmented regression and limited cubic spline models were used in the investigation to characterize the threshold effects and non-linear correlations. Additionally, subgroup and interaction tests, Spearman correlation, least absolute shrinkage, and selection operator regression studies were conducted.

**Results:**

The 3047 participants included in this study had a mean age of 58.63 years and 51.79% were female. After fully adjusting for all covariates, the ln-SIRI (OR 1.39 [CI 1.10–1.74], P = 0.005), ln-AISI (OR 1.26 [1.03–1.53], P = 0.024), and ln-MLR (OR 1.62 [1.15–2.30], P = 0.006) were significantly correlated with the odds of severe AAC. A non-linear dose-response relationship was observed between ln-SII and severe AAC. Additional subgroup analyses revealed that this relationship was more evident in the diabetic population. Additionally, MLR (AUC = 0.644) predicted the prevalence of severe AAC better than other biomarkers, and the prediction model constructed in conjunction with screened clinical indicators showed good predictive value (AUC = 0.853).

**Conclusions:**

In this study, we comprehensively evaluated and compared the associations between six biomarkers and severe AAC, and developed a clinical prediction model using the MLR with the best predictive effect. However, cohort studies and model validation are still needed in the future to further confirm their relationship.

## Introduction

Globally, cardiovascular disease (CVD) is the leading cause of death and has a significant influence on both worldwide health and economic systems. [1]. Abdominal aortic calcification (AAC), characterized by abnormal calcification in the walls of muscular or elastic arteries, is a common occurrence in the context of CVD [2]. Epidemiological evidence strongly links AAC with hypertension, diabetes mellitus, and other atherosclerotic conditions, severe AAC can induce hemodynamic alterations, heightening the risk of complications such as abdominal aortic aneurysms and other CVD [3]. Thus, diagnosing AAC is crucial for assessing overall cardiovascular risk and facilitating early interventions to mitigate the incidence of CVD. Currently, a lateral lumbar spine x-ray is a common and safe diagnostic method for AAC, effectively illustrating the extent and location of calcifications [4]. Additionally, the Kauppila AAC score has been devised to quantify the extent of AAC, thereby aiding clinical decision-making [4]. Although reliable diagnostic tools are available, further research is needed to assess risk factors for the development of severe AAC and to actively prevent it [5].

Inflammation is a complex physiological response designed to repair bodily damage involving various cells, including neutrophils, macrophages, lymphocytes, and platelets, alongside substances these cells produce [6]. Numerous studies have identified the inflammatory response is the initial catalyst for atherosclerosis, leading to endothelial dysfunction as well as the accumulation of inflammatory cells within the walls of blood vessels, which activates and promotes the release of cytokines and growth factors, enhancing the calcification process [7,8]. The interplay between inflammation and oxidative stress is also critical in promoting arterial calcification [9]. Thus, inflammation is recognized as a fundamental pathophysiological process in arterial calcification and plays a crucial role in the advancement of CVD.

Given the pivotal role that inflammation plays in the development of CVD, researchers have shown significant interest in identifying inflammatory biomarkers to assess arterial calcification, including monocyte-lymphocyte ratio (MLR), neutrophil-lymphocyte ratio (NLR), and platelet-lymphocyte ratio (PLR) [10–12]. These markers are essential for demonstrating the link between different symptoms of CVD and systemic inflammation [13,14]. Moreover, advancements have been achieved in creating more sophisticated and comprehensive indices, including systemic immune inflammation index (SII), systemic inflammatory response index (SIRI), and aggregate index of systemic inflammation (AISI). These three indices, which integrate four hematologic profiles, provide more accurate clinical insights compared to MLR, NLR, and PLR [15]. Previous research has validated the strong diagnostic and predictive capabilities of these immunoinflammatory markers in tumors and inflammation-related diseases. In contrast to complete blood counts, they can accurately characterize the body’s immune-inflammatory response, aid in the detection of inflammation, cancer, and arterial calcification, and have been shown to correlate with the development of most clinical diseases [16–19]. Despite these advances, there is still a lack of relevant studies to comprehensively assess the relationship between multiple systemic immunoinflammatory biomarkers and severe AAC and to compare their clinical predictive value. Therefore, our study aimed to comprehensively test the association of six systemic immunoinflammatory biomarkers (SII, SIRI, AISI, PLR, NLR, and MLR) with the likelihood of severe AAC and to explore the dose-response relationship between them and the differences between populations, and ultimately to assess the role of these biomarkers in predicting the severe AAC and construct a visualization model.

## Methods

### Study design and population

**Fig 1:**
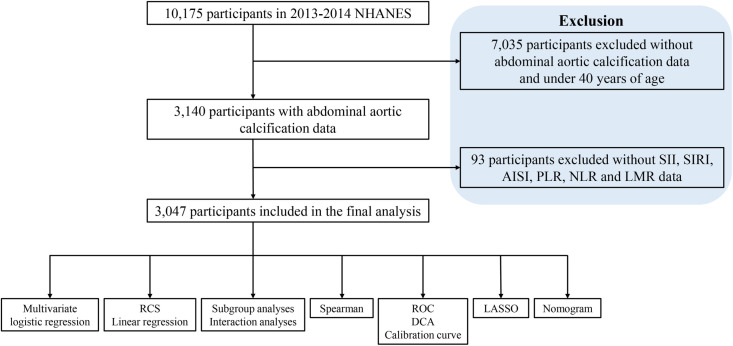
Flowchart of the participants’ selection from NHANES 2013–2014.

The NHANES is a nationally representative epidemiologic survey. It employs a complex multistage random sampling method to guarantee that it accurately represents the population [20]. During recruitment, the National Center for Health Statistics Research Ethics Review Committee approved the study methodology, and each participant signed a comprehensive written consent document [21]. We collected data on participants’ AAC scores during the 2013–2014 period, as well as other important health indicators, including BMI, waist circumference, grip strength, and disease status. During the data processing stage, we excluded 7,035 individuals with absent AAC data and 93 individuals with incomplete information on neutrophils, monocytes, lymphocytes, and platelets. In the completed analysis, Fig 1 displays that 3,047 individuals were deemed eligible and subsequently included.

Definition of systemic immunoinflammatory biomarkers (SII, SIRI, AISI, PLR, NLR, MLR)

The NHANES database provides the essential data required for calculating systemic immunoinflammatory biomarkers. These counts underwent rigorous laboratory testing and are reported in units of × cells/μl. Using the results from these complete blood counts, we derived six systemic immunoinflammatory biomarkers with the following formulas [18]:

SII = platelet × neutrophil/lymphocyte count;

SIRI = monocyte × neutrophil/lymphocyte count;

AISI = neutrophil × platelet× monocyte/lymphocyte count;

PLR = platelet/lymphocyte count;

NLR = neutrophil/lymphocyte count;

MLR = monocyte/lymphocyte count.

### Evaluation of severe AAC

To assess AAC, the Kauppila scoring system was utilized via dual energy X-ray absorptiometry (DXA) [21]. DXA is a method that calculates the bone mineral content and soft tissue composition of a scanned region based on the differential attenuation of high and low energy X-rays passing through the body. When evaluating AAC, DXA specifically targets the lumbar spine region for lateral scans, covering the vertebrae from L1-L4 [22–24], to quantitively evaluate the extent of AAC. In contrast, the Kauppila score method is mainly based on the midpoint of the intervertebral space as the boundary and divides the artery into anterior and posterior walls, and evaluates the calcification of the abdominal aorta in the vicinity of each vertebra separately, and the sum of the calcification scores of the anterior and posterior walls of the abdominal aorta in the vicinity of each vertebra is the total vascular calcification score [25,26]. The AAC score obtained by this method varies from 0 to 24, with elevated values representing greater calcification extent. In clinical and research settings, a threshold of AAC score ≥ 6 is commonly utilized to indicate severe calcification, signifying an elevated cardiovascular risk [27,28]. This scoring system, which can diagnose AAC with high specificity and sensitivity and expose subjects to lower radiation levels, has been recognized as a reliable method for assessing AAC [29,30].

### Covariate

Based on previous studies, our study comprehensively analyzed multiple covariates that could influence systemic immunoinflammatory biomarkers associated with AAC [11,31]. These covariates were categorized into several broad groups: demographic variables, physical examination variables, laboratory data variables and living health variables [32]. S1 Table lists the explanations and precise definitions of all covariates. In this case, data on poverty income ratios (PIR) were derived directly from gross annual household income reported in the NHANES questionnaire (INDFMPIR), and was categorized into three classes: low-income, < 1.3; middle-income, 1.3–3.5; and high-income, ≥ 3.5 [33,34]. Body mass index (BMI) is categorized through WHO international standards (2000) and previous studies as normal, < 25; overweight, 25–30; and obese, ≥ 30 [34,35]. Diabetes was defined as 1) self-reported diabetes mellitus; 2) insulin or glucose-lowering medications; 3) hemoglobin A1c (HbA1c) ≥ 6. 5%; and 4) fasting blood glucose (FBG) ≥ 7.0 mmol/L. Prediabetes mellitus was defined as 1) self-reported pre-diabetes mellitus; 2) HbA1c 5.7–6.4%; and 3) FBG 5.6–6.9 mmol/L [34,36]. Hypertension was defined as 1) self-reported hypertension; 2) use of antihypertensive medication; and 3) mean systolic blood pressure ≥ 140 mmHg or mean diastolic blood pressure ≥ 90 mmHg [37,38]. Hyperlipidemia was defined as 1) self-reported high cholesterol levels; 2) use of cholesterol-lowering medications; and 3) laboratory tests showing total cholesterol ≥ 200 mg/dl and high-density lipoprotein-cholesterol (HDL-C) < 40 mg/dl (male) or < 50 mg/dl (female). The definition of other diseases relies heavily on self-reported data. These self-reported medical histories were provided by trained participants and collected by specialized staff, ensuring their reliability and rigor in epidemiological applications, as has been validated in the reports of multiple studies [12,39]. Collection methods and details can be found at https://wwwn.cdc.gov/nchs/nhanes/.

### Statistical analysis

Baseline demographic data, represented by means (standard deviation) for normally distributed continuous variables; median (first quartile, third quartile) for skewed continuous variables; and frequencies (percentages) for categorical variables. Missing covariates were imputed using multiple imputations with five replications, employing a chained equation approach to account for missing data across replications [40]. Participants were categorized into severe AAC and non-severe AAC groups according to the Kauppila scoring system. Additionally, we assessed the normality of systemic immunoinflammatory biomarkers (SII, SIRI, AISI, PLR, NLR, and MLR) using both Kolmogorov-Smirnov tests and graphical methods by superimposing histograms with theoretical normal distribution curves. Since these data exhibited a non-normal distribution, a natural logarithm (ln) transformation was performed to achieve an approximate normal distribution (S2 Table and S1 Fig). Normally distributed continuous variables were analyzed using Student’s t-tests, skewed continuous variables were analyzed using Mann-Whitney U test, and categorical data were examined with Chi-square tests. In addition, all covariates involved in this study were tested for multicollinearity, and variables with high covariance were excluded to improve the stability of the model.

First, we utilized weighted multivariate logistic regression modeling to assess the correlation of SII, SIRI, AISI, PLR, NLR, and MLR with severe AAC, reporting the results as odds ratios (OR) with 95% confidence intervals (CI). Three distinct models were developed: a basic unadjusted model, model 2 with adjustments for all covariates identified, and model 1 with adjustments specifically for age, gender, and race. Since the effects of SII, AISI, and NLR on severe AAC were not significant for individual units, we explored the effects of SII, AISI, and NLR per 100 units on the potential for severe AAC in our analysis. Subsequently, the SII, SIRI, AISI, PLR, NLR, and MLR data were transformed into quartiles (Q1, Q2, Q3, and Q4), and the linear relationship between them and severe AAC was investigated using a trend test [14]. Second, we examined potential non-linear associations of these markers with severe AAC using restricted cubic spline (RCS) regression. RCS is able to capture the nonlinear trend of continuous variables (e.g., “U-shaped” and “J-shaped”) by flexibly setting the nodes, avoiding the bias of linear assumptions, and improving the model fitting effect and biological explanatory power. The reference point is set as the median, and the knots are determined according to the Minimizing Akaike Information Criterion (AIC). Upon detecting non-linearity, we calculated inflection points and employed segmented linear regression to analyze the threshold effects of these markers. Meanwhile, we performed subgroup analyses, interaction tests, and Spearman correlation analyses.

Moreover, least absolute contraction and selection operator (LASSO) regression were used to further screen the characteristic factors affecting AAC in the covariates. Additionally, the Receiver Operating Characteristic (ROC) curve was used to evaluate the model’s prediction ability for severe AAC. Finally, a comprehensive prediction model and nomogram were constructed. The validity of the model was assessed by ROC curve, decision curve analysis (DCA), and area under the curve (AUC). The nomogram depicted the correlation of overall scores with predicted prognosis. R software (version 4.3.3) was utilized to conduct every analysis. Statistical significance was determined with a p < 0.05 (2-sided).

## Results

### Baseline characteristics of participants

This study included 3,047 participants, as depicted in the screening flowchart (Fig 1). Table 1 presents the participants’ demographic characteristics along with other covariates. The groups are based on the presence of severe AAC. Interestingly, the participants’ average age was 58.63 years, 53.36% were under 60 years of age and 51.79% were female. Comparative analysis revealed that individuals with severe AAC tended to be of an advanced age, experienced greater incidences of smoking, and more frequently suffered from hypertension, hyperlipidemia, diabetes, CHD, myocardial infarction, stroke, and COPD, among other conditions.

**Table 1:** Baseline characteristics of subjects with and without severe AAC in NHANES 2013-2014.

Characteristic	Overall (n = 3047)	Non-Severe AAC (n = 2718)	Severe AAC (n = 329)	P value
Age (years)	58.63 ± 12.00	57.15 ± 11.39	70.91 ± 9.74	**<0.001**
Waist circumference (cm)	99.32 ± 13.58	99.44 ± 13.87	98.37 ± 10.81	0.101
Grip strength (kg)	68.10 ± 20.22	69.17 ± 20.24	59.24 ± 17.71	**<0.001**
Total cholesterol (mg/dL)	194.82 ± 42.88	196.10 ± 42.99	184.27 ± 40.50	**<0.001**
HDL-C (mg/dL)	54.06 ± 16.71	54.16 ± 16.79	53.26 ± 16.10	0.355
Vitamin D (nmol/L)	70.39 ± 29.24	69.29 ± 28.56	79.48 ± 33.01	**<0.001**
eGFR (mL/min/1.73m^2^)	83.85 ± 23.07	85.36 ± 22.39	71.32 ± 24.76	**<0.001**
SII	444.00 (316.88, 622.20)	439.23 (314.20, 609.48)	492.77 (347.14, 734.34)	**<0.001**
SIRI	1.08 (0.73, 1.60)	1.06 (0.72, 1.53)	1.46 (0.92, 2.12)	**<0.001**
AISI	242.31 (156.28, 372.57)	236.49 (154.03, 356.86)	315.73 (187.20, 483.60)	**<0.001**
PLR	2.00 (1.48, 2.64)	1.95 (1.47, 2.55)	2.33 (1.68, 3.25)	**<0.001**
NLR	113.81 (89.26, 143.08)	113.57 (89.42, 142.13)	115.88 (89.00, 147.00)	0.199
MLR	0.28 (0.21, 0.35)	0.27 (0.21, 0.34)	0.33 (0.26, 0.43)	**<0.001**
Age, n (%)	58.63 ± 12.00	57.15 ± 11.39	70.91 ± 9.74	**<0.001**
<60 years	1626 (53.36)	1579 (58.09)	47 (14.29)	
≥60 years	1421 (46.64)	1139 (41.91)	282 (85.71)	
Gender, n (%)				0.943
Male	1469 (48.21)	1311 (48.23)	158 (48.02)	
Female	1578 (51.79)	1407 (51.77)	171 (51.98)	
Race/ethnicity, n (%)				**<0.001**
Mexican American	402 (13.19)	373 (13.72)	29 (8.81)	
Other Hispanic	289 (9.48)	273 (10.04)	16 (4.86)	
Non-Hispanic White	1349 (44.27)	1143 (42.05)	206 (62.61)	
Non-Hispanic Black	586 (19.23)	541 (19.90)	45 (13.68)	
Other Race	421 (13.82)	388 (14.28)	33 (10.03)	
PIR, n (%)				0.143
<1.3	845 (27.73)	748 (27.52)	97 (29.48)	
1.3-3.5	1213 (39.81)	1072 (39.44)	141 (42.86)	
≥3.5	989 (32.46)	898 (33.04)	91 (27.66)	
Education level, n (%)				0.358
Less than 12th grade	700 (22.97)	615 (22.63)	85 (25.84)	
High school or equivalent	688 (22.58)	606 (22.30)	82 (24.92)	
Some college or AA degree	857 (28.13)	771 (28.37)	86 (26.14)	
College graduate or above	800 (26.26)	724 (26.64)	76 (23.10)	
Rejection and missing	2 (0.07)	2 (0.07)	0 (0.00)	
BMI, n (%)				**<0.001**
<25 kg/m2	866 (28.42)	758 (27.89)	108 (32.83)	
25-30 kg/m2	1111 (36.46)	967 (35.58)	144 (43.77)	
≥30 kg/m2	1070 (35.12)	993 (36.53)	77 (23.40)	
Smoking status, n (%)				**<0.001**
Yes	1406 (46.14)	1209 (44.48)	197 (59.88)	
No	1641 (53.86)	1509 (55.52)	132 (40.12)	
Alcohol consumption, n (%)				0.290
Yes	2042 (67.02)	1813 (66.70)	229 (69.60)	
No	1005 (32.98)	905 (33.30)	100 (30.40)	
Hypertension, n (%)				**<0.001**
Yes	1694 (55.60)	1428 (52.54)	266 (80.85)	
No	1353 (44.40)	1290 (47.46)	63 (19.15)	
Hyperlipidemia, n (%)				**<0.001**
Yes	2247 (73.74)	1972 (72.55)	275 (83.59)	
No	800 (26.26)	746 (27.45)	54 (16.41)	
Diabetes, n (%)				**<0.001**
Yes	671 (22.02)	549 (20.20)	122 (37.08)	
No	1254 (41.16)	1167 (42.94)	87 (26.44)	
Prediabetes	1122 (36.82)	1002 (36.87)	120 (36.47)	
CHD, n (%)				**<0.001**
Yes	159 (5.22)	98 (3.61)	61 (18.54)	
No	2888 (94.78)	2620 (96.39)	268 (81.46)	
Myocardial infarction, n (%)				**<0.001**
Yes	151 (4.96)	105 (3.86)	46 (13.98)	
No	2896 (95.04)	2613 (96.14)	283 (86.02)	
Stroke, n (%)				**<0.001**
Yes	131 (4.30)	95 (3.50)	36 (10.94)	
No	2916 (95.70)	2623 (96.50)	293 (89.06)	
COPD, n (%)				**<0.001**
Yes	129 (4.23)	99 (3.64)	30 (9.12)	
No	2918 (95.77)	2619 (96.36)	299 (90.88)	
Cancer, n (%)				**<0.001**
Yes	380 (12.47)	300 (11.04)	80 (24.32)	
No	2667 (87.53)	2418 (88.96)	249 (75.68)	
Hypoglycemic therapy, n (%)				**<0.001**
Yes	450 (14.77)	364 (13.39)	86 (26.14)	
No	2597 (85.23)	2354 (86.61)	243 (73.86)	
Cholesterol-lowering therapy, n (%)				**<0.001**
Yes	839 (27.54)	665 (24.47)	174 (52.89)	
No	2208 (72.46)	2053 (75.53)	155 (47.11)	
Antihypertensive therapy, n (%)				**<0.001**
Yes	1144 (37.55)	927 (34.11)	217 (65.96)	
No	1903 (62.45)	1791 (65.89)	112 (34.04)	

Normally distributed continuous variables: values are expressed as mean ± standard deviation.

Skewed distributions continuous variables: values are expressed as median (first quartile, third quartile).

Categorical variables: values are expressed as numbers (percentage).

Abbreviation: AAC, abdominal aortic calcification; eGFR, estimated glomerular filtration rate; PIR, poverty income ratio; BMI, body mass index; CHD, coronary heart disease; COPD, chronic obstructive pulmonary disease; HDL-C, high-density lipoprotein cholesterol; SII, systemic immune-inflammation index; SIRI, system inflammation response index; AISI, aggregate index of systemic inflammation; PLR, platelet-to-lymphocyte ratio; NLR, neutrophil-to-lymphocyte ratio; MLR, Monocyte-to-lymphocyte ratio.

### Assessment of multicollinearity of variables

We assessed multicollinearity among covariates using variance inflation factor (VIF) analysis. The results showed that the VIF values were below 5 for all variables except BMI and waist circumference (S3 Table). To be precise, a VIF value equal to 1 indicates that there is no multicollinearity between the factors; between 1 and 5 indicates that there may be correlation but it is not a cause for undue concern; between 5 and 10 indicates that there may be a high degree of correlation; and greater than 10 can be considered as too high a degree of multicollinearity and the model is relatively unstable [34]. The above results suggest that multicollinearity exists between BMI and waist circumference in the model of this study, which prompted us to exclude the variable of waist circumference, considering the central position of the BMI index in the assessment system and its higher clinical applicability. Subsequent VIF analyses showed that there was no significant multicollinearity in this study’s model (S3 Table).

### Correlation of odds of severe AAC with systemic immunoinflammatory biomarkers (SII, SIRI, AISI, PLR, NLR, and MLR)

Table 2 summarizes the results of our analysis using multivariate logistic regression. In the unadjusted and minimally adjusted models, increases in the ln-transformed values of SII, SIRI, AISI, PLR and MLR were significantly correlated with a higher likelihood of severe AAC. However, when the model was thoroughly adjusted, there was no statistically significant correlation between ln-SII and ln-PLR and severe AAC. Conversely, the relationships with ln-SIRI (OR 1.39 [CI 1.10–1.74], P = 0.005), ln-AISI (OR 1.26 [1.03–1.53], P = 0.024), and ln-MLR (OR 1.62 [1.15–2.30], P = 0.006) remained significant. Specifically, a one-unit increase in ln-SIRI, ln-AISI, and ln-MLR corresponded to a 39%, 26%, and 62% greater odds of severe AAC. This trend persisted in further sensitivity analyses, where we categorized systemic immunoinflammatory biomarkers into quartiles. When fully adjusted for all covariates, the difference between the highest quartile’s (Q4) and lowest quartile’s (Q1) odds of severe AAC increased by 55% for SII, 85% for SIRI, 62% for AISI, 56% for PLR, and 65% for MLR (P < 0.05).

**Table 2:** Association between systemic immune-inflammatory biomarkers (SII, SIRI, AISI, PLR, NLR, MLR) and severe AAC.

Characteristic		Crude model ^a^		Model 1 ^b^		Model 2 ^c^	
**OR (95% CI)**	**P value**	**OR (95% CI)**	**P value**	**OR (95% CI)**	**P value**
**Ln-SII**		1.54 (1.25, 1.91)	<0.001	1.31 (1.05, 1.65)	0.019	1.21 (0.95, 1.54)	0.128
**SII/100** **Quartile**	Q1	Ref		Ref		Ref	
	Q2	1.08 (0.77, 1.53)	0.653	1.11 (0.76, 1.62)	0.578	1.17 (0.79, 1.73)	0.436
	Q3	0.99 (0.69, 1.41)	0.941	0.93 (0.63, 1.37)	0.719	0.92 (0.61, 1.38)	0.688
	Q4	1.92 (1.40, 2.63)	<0.001	1.61 (1.13, 2.28)	0.008	1.55 (1.07, 2.26)	**0.022**
	P for trend		<0.001		0.004		**0.048**
**Ln-SIRI**		2.19 (1.82, 2.63)	<0.001	1.56 (1.26, 1.93)	<0.001	1.39 (1.10, 1.74)	**0.005**
**SIRI** **Quartile**	Q1	Ref		Ref		Ref	
	Q2	1.69 (1.13, 2.52)	0.011	1.25 (0.82, 1.92)	0.298	1.28 (0.82, 1.99)	0.281
	Q3	2.03 (1.37, 3.01)	<0.001	1.47 (0.97, 2.24)	0.071	1.51 (0.97, 2.34)	0.068
	Q4	3.99 (2.77, 5.74)	<0.001	2.16 (1.45, 3.23)	<0.001	1.85 (1.20, 2.83)	**0.005**
	P for trend		<0.001		<0.001		**0.003**
**Ln-AISI**		1.64 (1.39, 1.94)	<0.001	1.37 (1.14, 1.65)	<0.001	1.26 (1.03, 1.53)	**0.024**
**AISI/100** **Quartile**	Q1	Ref		Ref		Ref	
	Q2	1.00 (0.69.1.45)	0.994	0.96 (0.65, 1.43)	0.845	0.95 (0.63, 1.45)	0.826
	Q3	1.29 (0.91, 1.84)	0.154	1.15 (0.79, 1.69)	0.462	1.14 (0.76, 1.70)	0.534
	Q4	2.36 (1.71, 3.26)	<0.001	1.79 (1.25, 2.56)	0.001	1.62 (1.10, 2.38)	**0.014**
	P for trend		<0.001		<0.001		**0.006**
**Ln-PLR**		2.32 (1.83, 2.96)	<0.001	1.48 (1.14, 1.92)	0.004	1.32 (1.00, 1.74)	0.051
**PLR** **Quartile**	Q1	Ref		Ref		Ref	
	Q2	1.18 (0.81, 1.72)	0.384	1.17 (0.78, 1.75)	0.456	1.19 (0.78, 1.82)	0.411
	Q3	1.50 (1.05, 2.15)	0.026	1.24 (0.84, 1.83)	0.271	1.24 (0.83, 1.86)	0.295
	Q4	2.64 (1.90, 3.69)	<0.001	1.64 (1.14, 2.37)	0.008	1.56 (1.01, 2.19)	**0.044**
	P for trend		<0.001		0.005		**0.049**
**Ln-NLR**		1.26 (0.93, 1.70)	0.143	1.04 (0.76, 1.43)	0.801	1.12 (0.80, 1.56)	0.513
**NLR/100** **Quartile**	Q1	Ref		Ref		Ref	
	Q2	0.85 (0.61, 1.19)	0.352	0.81 (0.56, 1.16)	0.242	0.92 (0.63, 1.35)	0.681
	Q3	0.96 (0.69, 1.32)	0.790	0.96 (0.67, 1.37)	0.825	1.05 (0.72, 1.54)	0.800
	Q4	1.15 (0.84, 1.57)	0.384	0.94 (0.67, 1.34)	0.744	1.08 (0.74, 1.57)	0.691
	P for trend		0.229		0.977		0.561
**Ln-MLR**		3.61 (2.72, 4.80)	<0.001	1.71 (1.23, 2.37)	0.002	1.62 (1.15, 2.30)	**0.006**
**MLR** **Quartile**	Q1	Ref		Ref		Ref	
	Q2	1.57 (1.05, 2.36)	0.028	1.29 (0.84, 1.99)	0.243	1.30 (0.83, 2.04)	0.252
	Q3	2.01 (1.35, 2.99)	<0.001	1.30 (0.85, 2.01)	0.230	1.40 (0.89, 2.19)	0.143
	Q4	3.80 (2.64, 5.48)	<0.001	1.73 (1.14, 2.61)	0.010	1.65 (1.07, 2.55)	**0.023**
	P for trend		<0.001		0.007		**0.027**

In sensitivity analysis, SII/100, SIRI, AISI/100, PLR, NLR/100, MLR was converted from a continuous variable to a categorical variable (quartile).

OR: odds ratio.

95% CI: 95% confidence interval.

^a^ Crude model: no covariates were adjusted.

^b^ Model 1: adjusted for gender, age, and race.

^c^ Model 2: gender, age, race, PIR, education level, BMI, smoking status, alcohol consumption, grip strength, total cholesterol, HDL-C, Vitamin D, eGFR, hypertension, hyperlipidemia, diabetes, CHD, myocardial infarction, stroke, COPD, cancer, hypoglycemic therapy, cholesterol-lowering therapy, antihypertensive therapy.

### Dose-response relationship regarding severe AAC odds and systemic immunoinflammatory biomarkers (SII, SIRI, AISI, PLR, NLR, MLR)

**Fig 2:**
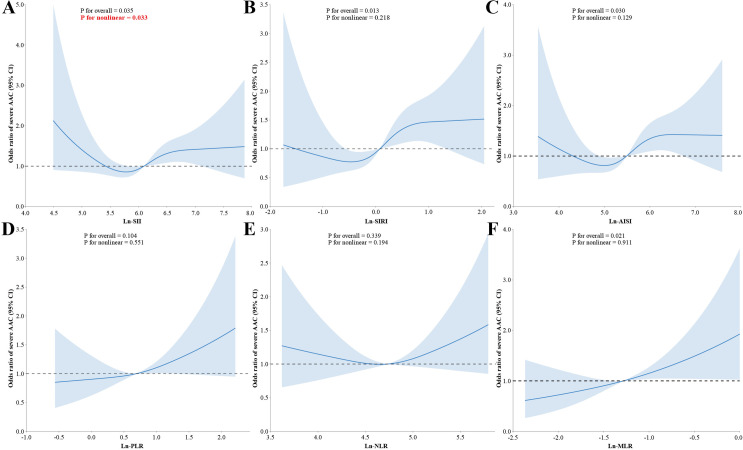
Dose-response relationships between ln-transformed SII, SIRI, AISI, PLR, NLR, MLR, and severe AAC. **(A)** Dose-response of lnSII and severe AAC; **(B)** Dose-response of lnSIRI and severe AAC; **(C)** Dose-response of lnAISI and severe AAC; **(D)** Dose-response of lnPLR and severe AAC; **(E)** Dose-response of lnNLR and severe AAC; and **(F)** Dose-response of lnMLR and severe AAC. SII, systemic immune-inflammation index; SIRI, systemic inflammation response index; AISI, aggregate index of systemic inflammation; PLR, platelet- to-lymphocyte ratio; NLR, neutrophil-to-lymphocyte ratio; MLR, monocyte-to-lymphocyte ratio.

The results of the RCS regression model are depicted in Fig 2. All RCS models were fitted with four knots according to the minimized AIC. After controlling for all confounding variables, we observed a non-linear dose-response relationship for SII, indicating a significant non-linear association with severe AAC (P < 0.05 for non-linearity). As detailed in Table 3, the outcomes from the segmented linear regression indicate a critical inflection point for ln-transformed SII at 5.27. Below the inflection point, ln-SII was not significantly related to the odds of severe AAC. Conversely, above an ln-SII value of 5.27, the likelihood of severe AAC rose by 41% (OR 1.41 [1.08–1.84], P = 0.012) (the log-likelihood test is 0.015). This suggests that the higher the level of SII, the greater the odds of severe AAC occurring.

**Table 3:** Two-piecewise linear regression modeling to analyze the threshold effect of lnSII on severe AAC.

Adjusted Models ^a^	OR (95% CI)	P value
**Model I**		
Standard linear model	1.19 (0.96,1.50)	0.151
**Model II**		
Two-piecewise linear regression		
Inflection point	5.27	
<5.27	0.38 (0.14,1.04)	0.119
≥5.27	1.41 (1.08,1.84)	**0.012**
Log likelihood ratio ^b^		**0.015**

OR: odds ratio.

95% CI: 95% confidence interval.

^a^ Adjusted models: gender, age, race, PIR, education level, BMI, smoking status, alcohol consumption, grip strength, total cholesterol, HDL-C, Vitamin D, eGFR, hypertension, hyperlipidemia, diabetes, CHD, myocardial infarction, stroke, COPD, cancer, hypoglycemic therapy, cholesterol-lowering therapy, antihypertensive therapy.

^b^ Model II compared to model I.

### Subgroup and interaction analyses

**Fig 3:**
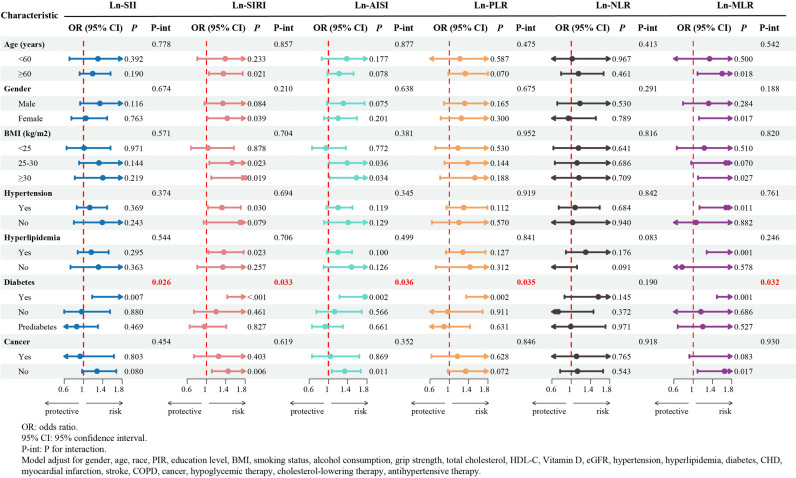
Subgroup and interaction analysis of the association between systemic immunoinflammatory biomarkers (SII, SIRI, AISI, PLR, NLR, MLR) and severe AAC.

As detailed in Fig 3, we categorized the participants according to age, gender, BMI, and health conditions, and conducted multiple logistic regression analyses. In most subgroups, ln-SII, ln-SIRI, ln-AISI, ln-PLR, ln-NLR, and ln-MLR did not interact significantly with severe AAC (P for interaction > 0.05). However, there was a significant interaction between the biomarkers and severe AAC in the diabetes subgroup, except for ln-NLR (P for interaction < 0.05). Specifically, within the diabetic cohort, for each one-unit increase in ln-transformed SII, SIRI, AISI, PLR, and MLR, the odds of severe AAC rose significantly by 84%, 115%, 76%, 121%, and 175% respectively (S4 Table). These findings emphasize that diabetes may be a key modifier of the association of inflammation with AAC.

### Correlation of systemic immunoinflammatory biomarkers with abdominal aortic calcification of participants

The relationship between systemic immunoinflammatory biomarkers, AAC scores, and the prevalence of severe AAC in research participants was evaluated using Spearman correlation analysis. All of the aforementioned indices were statistically significant with regard to the AAC score and the occurrence of severe AAC (p < 0.05), as shown in S5 Table. Notably, MLR exhibited the strongest positive correlation with AAC scores (R = 0.128, P < 0.001) and severe AAC (R = 0.154, P < 0.001), indicating a robust association. In addition, there appeared to be very weak negative and positive correlations between NLR and AAC scores (R = −0.021, P = 0.004) and severe AAC (R = 0.023, P = 0.002), respectively.

### Diagnostic power of different systemic immunoinflammatory biomarkers for severe AAC

**Fig 4:**
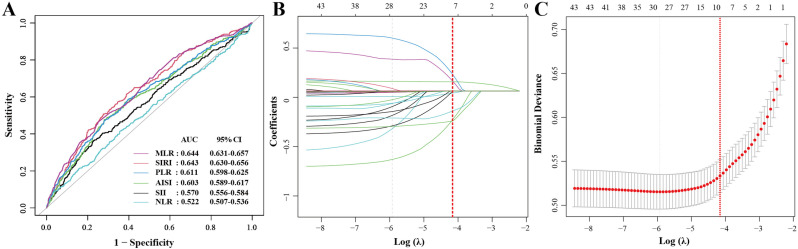
(A) Comparison of predictive values of SII, SIRI, AISI, PLR, NLR, and MLR for the prevalence of severe AAC; (B) Regression coefficient profile when using LASSO regression for feature screening of severe AAC prediction. Each curve represents the change trajectory of each feature coefficient; (C) Cross-validation curve of LASSO regression. Each red dot represents the mean square error (MSE) for each value. The ordinate is the value of the coefficient, the abscissa (top) is the number of non-zero coefficients in the model, and the abscissa (bottom) is the logarithm of the regularization parameter . The dashed line on the right shows the optimal value of lambda (best = −4.157). For the prediction of severe AAC, we used for variable screening. Eight variables were screened, including age, race, BMI, smoking status, CHD, diabetes, antihypertensive therapy, cholesterol-lowering therapy.

As illustrated in Fig 4A, we employed ROC curve analyses to assess the effectiveness of multiple systemic immunoinflammatory biomarkers in identifying participants with severe AAC, including SII, SIRI, AISI, PLR, NLR, and MLR. The results indicated that MLR, with an AUC value of 0.644 (95% CI 0.631–0.657, P < 0.001) and an optimum cutoff level of 0.344, a sensitivity of 0.749 (95% CI 0.732–0.765), and a specificity of 0.465 (95% CI 0.411–0.519), was particularly effective in identifying severe AAC (S6 Table). Among them, the optimal cutoff value was determined by maximizing the Youden index.

### LASSO regression analysis and construction of the final model

**Fig 5:**
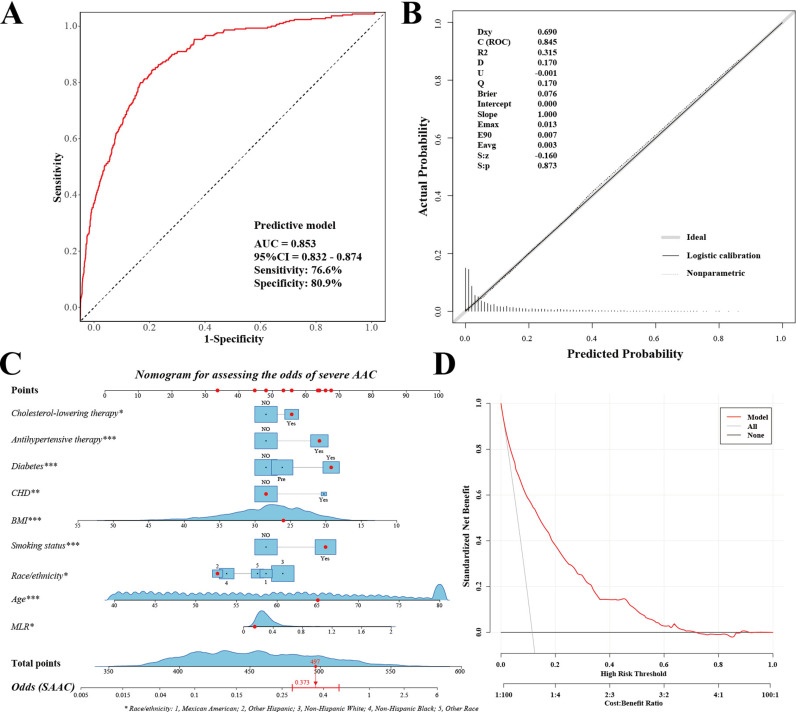
Performance evaluation of the severe AAC prediction model and construction of a nomogram. **(A)** ROC curve. The AUC of the model was 0.853 (95% CI = 0.832-0.874). The specificity of the model was 80.9% and the sensitivity was 76.6%; **(B)** Calibration curve. The solid gray line (Ideal) represents the calibration curve for the ideal prediction model, the solid black line (Logistic calibration) represents the prediction model calibrated using logistic regression, and the dashed gray line (Nonparametric) represents the actual prediction model; **(C)** Nomogram. The top horizontal line is the score column, and the total score based on the item-by-item sum of the scores for each item is the probability of severe AAC. Taking the red dot as an example, the participants in this state are 37.3% more likely to suffer from AAC; **(D)** Decision curve analysis (DCA). The red line indicates the net benefit of using the predictive model. The black line indicates if the predictive model is not used.

Additionally, we further refined our selection of clinical variables for the final prediction model using LASSO regression analysis, identifying 8 key factors (S7 Table and Fig 4B-C). As depicted in Fig 5A, ultimately, by results from LASSO regression analyses, we constructed a final prediction model incorporating 9 variables. This model demonstrated robust predictive performance, evidenced by a high AUC value of 0.853 (95% CI 0.8320.874), the model was 76.6% sensitive and 80.9% specific. Furthermore, the average AUC value of the predictive model was 0.847 based on 10-fold cross-validation, which was consistent with the previously constructed value (S2 Fig). Further DCA showed that the model possesses a high decision value (Fig 5D). Meanwhile, AUC of 0.845, Brier value of 0.076 (<0.25), and P = 0.873 (>0.05) are also shown by the calibration curve in Fig 5B, suggesting that this predictive model has strong predictive ability and that the fitted line agrees well with the standard reference line.

### Construction of the nomogram

Subsequently, we developed a nomogram utilizing the nine clinical variables identified earlier, assigning a score to each variable. The cumulative total score from these variables can indicate a subject’s likelihood of developing severe AAC. This approach enhances the visualization and facilitates more precise predictions of the odds associated with developing severe AAC, as depicted in Fig 5C.

## Discussion

Our research represents the initial cross-sectional analysis to simultaneously evaluate the predictive capability of six systemic immunoinflammatory biomarkers for severe AAC. Among the 3,047 participants included in our study, we observed several key findings: 1) SII, SIRI, AISI, PLR, and MLR were positively correlated with severe AAC, suggesting that an increased odds of severe AAC may be linked to higher levels of systemic inflammation; 2) A non-linear dose-response relationship was identified linking ln-SII with severe AAC, the inflection point occurred at ln-SII = 5.27, above which a positive correlation was evident; 3) Subgroup and interaction analyses indicated significant differences and interactions in the associations between SII, SIRI, AISI, PLR, MLR, and severe AAC among diabetic and non-diabetic participants; 4) ROC curve analysis demonstrated that MLR may be especially helpful in anticipating the odds of severe AAC.

Chen et al. conducted a notable study in this area, demonstrating that SII, NLR, and PLR are independent predictors of colorectal cancer, and that SII is particularly effective in differentiating patients with similar TNM staging but very high risk [41]. Wang et al. explored the relationship between SIRI and breast cancer, finding that patients with a SIRI < 0.65 exhibited higher overall survival rates, particularly in post-operative patients with breast cancer [42]. Concurrently, Wang et al. assessed the relationship between systemic immunoinflammatory biomarkers and prostate cancer, discovering that MLR was more sensitive and accurate in distinguishing the presence of prostate cancer [43]. Li et al. further expanded the scope of their research by exploring the connection between inflammatory states and non-cancerous diseases, particularly by comparing the levels of SII, SIRI, NHR (neutrophil-to-hemoglobin ratio), LHR (lymphocyte-to-hemoglobin ratio), MHR (monocyte-to-hemoglobin ratio), and PHR (platelet-to-hemoglobin ratio) with chronic kidney disease (CKD). They proposed that elevated levels of SIRI, SII, NHR, MHR, and PHR may increase the potential odds of developing CKD and emphasized that SIRI might be a better biomarker for predicting CKD [44]. Zhang et al. identified a potential association between PLR and MLR and primary membranous nephropathy (PMN), noting that PMN patients with poor treatment outcomes often exhibit higher PLR levels, and elevated MLR levels are associated with adverse renal prognosis [45]. Regarding nephrotic syndrome, Toraman and his team also confirmed the relationship between NLR and PLR and rapidly progressive glomerulonephritis (RPGN). They found that fibrinoid necrosis was positively correlated with PLR, and negatively correlated with lymphocyte count and hematocrit, concluding that the NLR-PLR marker can be used for grading glomerular inflammation and serves as a relatively accurate, economical new indicator for predicting RPGN clinical challenges [46]. Similarly, systemic immunoinflammatory biomarkers have been found to be closely related to the occurrence and progression of kidney stones. Mao et al.’s cross-sectional study indicated that higher levels of NLR are positively correlated with the prevalence of kidney stones and the number of stones excreted, suggesting that NLR, which can be easily obtained from blood tests, could serve as a novel indicator for kidney stone prevention and screening [47]. Furthermore, Tung et al. explored the association between NLR and both uric acid stones and CKD, demonstrating that patients with higher NLR levels, especially those with uric acid stones, are more likely to progress to CKD in the later stages [48]. In liver diseases, Zhao et al. examined the connection between SII and Non-alcoholic fatty liver disease (NAFLD), uncovering a “J”-shaped non-linear association of with mortality [49]. These findings underscore that the values of systemic immunoinflammatory biomarkers are closely related to the severity across various non-neoplastic diseases, broadening their potential applications.

Current research robustly supports the notion that inflammation is fundamental to the pathogenesis of vascular calcification [50,51]. This is primarily due to the inflammation’s regulation of vascular smooth muscle cell expression, which promotes calcification [52,53]. Specifically, monocytes are crucial in the early stages of arterial calcification; they migrate from the bloodstream to the vessel wall, influenced by chemokines, and later differentiate into macrophages [54]. These macrophages contribute to arterial calcification through producing various cytokines and matrix-degrading enzymes [55]. Neutrophils, which are abundant in the body, contribute to inflammation and calcium deposition in the arterial wall by oxidizing and degrading lipids and proteins through the release of oxidative stress product and proteases, further promoting the formation of calcified plaques [10]. Additionally, platelets serve a dual function in arterial calcification: they not only attach to the vessel wall, promoting the formation of atheromatous plaques [56], but their activation also exacerbates the inflammatory response and facilitates thrombosis, ultimately driving vascular calcification [57]. In contrast, lymphocytes may offer protective effects against arterial calcification by releasing anti-inflammatory and immunosuppressive factors, thus inhibiting excessive inflammatory responses and preventing further deterioration [58]. This aligns with the non-linear dose-response relationship between SII and severe AAC revealed in our study, suggesting that excessively high SII levels are associated with an increased odds of severe AAC. When SII is elevated, it may be due to an increase in platelet and neutrophil counts, leading to the release of a large amount of inflammatory mediators, chemokines, and reactive oxygen species, which adhere to the vessel wall, induce endothelial cell damage, and cause tissue ischemia, ultimately leading to adverse outcomes [59]. Moreover, elevated SII may also be attributed to a reduction in lymphocyte levels, as low lymphocyte counts weaken the suppression of endothelial cell inflammation, promoting the progression of atherosclerosis and vascular calcification [60]. Notably, beyond blood markers, a higher dietary inflammatory index was consistently linked with an increased AAC odds, displaying consistent trends across various populations. [61].

The relationships of SII, SIRI, AISI, PLR, and MLR with the prevalence for severe AAC after ln conversion were substantially different in diabetic and non-diabetic populations, according to additional subgroup analyses. This difference may be attributed to several key factors: 1) Diabetes, particularly type 2 diabetes, often induces a chronic, low-grade systemic inflammation. The hyperglycemic state activates multiple inflammatory pathways, such as nuclear factor kappa-B and NOD-like receptor thermal protein domain associated protein 3, which accelerate the production of inflammatory mediators like tumor necrosis factor-α, interleukin-6, and C-reactive protein [62]. These mediators contribute to endothelial cell damage and dysfunction, thereby accelerating the process of arterial calcification [63]. 2) Diabetic patients experience significant alterations in lipid metabolism and vascular function, characterized by increased low-density lipoprotein cholesterol and other components of non-high-density lipoprotein cholesterol [64]. The accumulation of lipids promotes atherosclerosis, leading to endothelial dysfunction, such as decreased nitric oxide synthesis and smooth muscle cell proliferation, ultimately influencing the progression of arterial calcification [65]. 3) Insulin resistance, a common feature in diabetes, leads to increased production of very-low-density lipoprotein (VLDL) by the liver. VLDL, through the enterohepatic circulation, causes hyperlipidemia, which raises levels of small, dense LDL, thereby increasing endothelial permeability, promoting monocyte-macrophage aggregation, and accelerating the formation of atherosclerotic plaques and calcification [34]. 4) Although the use of glucose-lowering or insulin medications improves insulin resistance and ameliorates vascular inflammation and arterial calcification, long-term use of such medications, especially sodium-glucose cotransporter 2 inhibitors and sulfonylureas, may induce local inflammation, thus influencing the progression of arterial calcification [66]. 5) Diabetes is often associated with bone metabolism disorders, with studies showing lower serum osteocalcin levels in diabetic patients, which may be closely linked to arterial calcification [67,68]. 6) Diabetes frequently coexists with multiple comorbidities, and the combined effects of these diseases may exacerbate the impact on arterial calcification [69,70]. However, due to the lack of specific classification of diabetes types and the absence of disease progression tracking and adjustment in the NHANES database, the association between diabetes and severe AAC still requires further investigation. Notably, this study observed a weak negative correlation between NLR and AAC (R = −0.021, P = 0.004) and a weak positive correlation with severe AAC (R = 0.023, P = 0.002). However, no significant association was found in both multivariate logistic regression and RCS analyses, suggesting that this weak correlation may lack biological significance. A multicenter study by Ban et al. demonstrated a significant association between NLR and AAC in patients with end-stage renal disease, but a similar report has not been seen in the general population [71]. This discrepancy suggests that the predictive value of NLR may be population-specific. Considering the large sample size of this study, these weak associations are more likely to be due to statistical noise. Future prospective studies with more rigorous design are needed to further validate the potential relationship between NLR and AAC in specific populations.

Given these insights, systemic immunoinflammatory biomarkers demonstrate substantial predictive value across various diseases. Since these biomarkers can be derived from routine cell count tests, which are low-cost and convenient, hold promising prospects for future clinical applications. Our study possesses several strengths. First, we utilized participants from the NHANES database, which furnishes a substantial and geographically representative sample. Second, we conducted subgroup analyses on various clinical indicators to affirm the robustness of the associations between systemic immunoinflammatory biomarkers and severe AAC in diabetic and non-diabetic populations. Third, we explored the threshold effect of SII on severe AAC using RCS curves and further performed segmented logistic regression. Moreover, while systemic immunoinflammatory biomarkers have often been analyzed as single indicators in previous studies, we compared the associations between the six systemic immunoinflammatory biomarkers and severe AAC more comprehensively, which has not been explored by researchers in this field. Finally, we preliminarily assessed the ability of the six systemic immunoinflammatory biomarkers to predict severe AAC using ROC curves, and constructed a more complete clinical prediction model and nomogram by further LASSO feature screening.

However, there are several limitations to our study. First, as a cross-sectional study utilizing the NHANES database, it cannot establish causality between systemic immunoinflammatory biomarkers and severe AAC; longitudinal cohort studies are required to elucidate this relationship. Second, the accuracy of medical history information in the NHANES database relies on self-reported questionnaires, data on AAC are limited to those aged 40 years and older who were included from 2013 to 2014, and some covariates are missing more severely, which could affect the control over diagnoses and the authenticity of disease extent. Third, as the NHANES dataset originates from a cross-sectional survey of the non-institutionalized U.S. population, it may not include hospitalized patients, individuals with severe diseases, or residents from certain geographic regions. Therefore, our study may not fully reflect the true situation of all affected populations, especially those with severe comorbidities or undergoing treatment, which may limit the generalizability of the results, particularly when assessing association for extreme or severely ill patients, leading to potential selection bias. Additionally, the voluntary nature of participation may result in underrepresentation of certain groups, such as low-income populations or older adults. Fourth, although multiple relevant correlations were considered, it is not possible to completely eliminate the influence of all potential factors, such as autoimmune diseases, genetic conditions, and hereditary disorders. Fifth, considering that our study sample is primarily derived from the U.S. population, with the majority being Mexican Americans, non-Hispanic Whites, and non-Hispanic Blacks, the applicability of the findings to other countries or regions may be limited. Finally, this study established an uncomplicated model to forecast the onset of severe AAC, but it lacked a professional model validation method, and its predictive value needs further validation. Given these considerations, future prospective, multi-center studies are needed to include samples from a wider range of ethnicities to further explore the causal relationship between systemic immunoinflammatory biomarkers and severe AAC prevalence. Additionally, incorporating more samples and adopting more specialized and comprehensive methods to refine our model will be crucial to ensure its generalizability and clinical value across different populations.

## Conclusion

Our study identified a significant relationship linking SII, SIRI, AISI, PLR, MLR to severe AAC odds. The combination of these clinical indicators not only helps to develop models for predicting severe AAC in the clinic, but also could aid in early diagnosis and personalized treatment. In the future, a comprehensive assessment combining these biomarkers may aid in clinical diagnosis and treatment, thus providing diabetic patients and other high-risk groups with more optimized management plans and improved cardiovascular health, and ultimately providing practical guidance for clinical practice.

## Supporting information

S1 Table All covariates and categorical definition of some of them. (DOCX)

S2 Table Kolmogorov-Smirnov tests for systemic immunoinflammatory biomarkers. (DOCX)

S3 Table Multicollinearity assessment of covariates. (DOCX)

S4 Table Subgroup and interaction analysis of the association between systemic immunoinflammatory biomarkers (SII, SIRI, AISI, PLR, NLR, MLR) and severe. AAC. (DOCX)

S5 Table Spearman correlation of systemic immunoinflammatory biomarkers with abdominal aortic calcification of participants. (DOCX)

S6 Table Diagnostic efficacy of systemic immunoinflammatory biomarkers (SII, SIRI, AISI, PLR, NLR, MLR) and constructed prediction model for severe. AAC. (DOCX)

S7 Table Lasso regression analysis results (best log(λ_1se_) = −4.157). (DOCX)

S1 Fig Histograms and theoretical normal curve overlays of systemic immunoinflammatory biomarkers. (A) SII and lnSII; (B) SIRI and lnSIRI; (C) AISI and lnAISI; (D) PLR and lnPLR; (E) NLR and lnNLR; (F) MLR and lnMLR.
(TIF)

S2 Fig ROC curves and area under the curve (95% CI) of the prediction model for 10-fold cross validation. (A) ROC curve for each fold; (B) average ROC curve for all folds.
(TIF)
