# Supplementary material for: Comparison of systemic immunoinflammatory biomarkers for assessing severe abdominal aortic calcification among US adults aged≥40 years: A cross-sectional analysis from NHANES
Source: PLoS One. 2025 Jun 24;20(6):e0325949. doi: 10.1371/journal.pone.0325949 (PMC12186907; doi:10.1371/journal.pone.0325949)
Supplement: S1 Table — (DOCX) [file pone.0325949.s001.docx]

**S1 Table** All covariates and categorical definition of some of them.

1 Data were obtained from the standardized variable INDFMPIR provided by NHANES. Referring to previous studies, PIR was classified into three levels: <1.3, 1.3-3.5, and ≥3.5.

2 Classification according to World Health Organization international standards (2000) and previous studies.

3 Calculated using the simplified modification of diet in renal disease (MDRD) formula, which is given as 186 * (serum creatinine)^-1.154^ * (age)^-0.203^ * 0.742 (female).

|  | **Covariates** | **Definition** |
| --- | --- | --- |
| **Demographic variables** | age | < 60 years and ≥ 60 years |
|  | gender | Male and female |
|  | race | Mexican American, Other Hispanic, On-Hispanic White, On-Hispanic Black, Other Race |
|  | poverty income ratio (PIR) ^1^ | Low-income, < 1.3; middle-income, 1.3-3.5; high-income, ≥ 3.5 |
|  | education level | Less than 12th grade, High school or equivalent, Some college or AA degree, College graduate or above, Rejection and missing |
| **Examination variables** | body mass index (BMI) ^2^ | Normal, <25 kg/m^2^; overweight, 25-30 kg/m^2^; obese, ≥30 kg/m^2^ |
|  | waist circumference (cm) | \ |
|  | grip strength (kg) | \ |
| **Laboratory variables** | total cholesterol (mg/dL) | \ |
|  | high-density lipoprotein cholesterol  (HDL-C, mg/dL) | \ |
|  | vitamin D (nmol/L) | \ |
|  | eGFR (mL/min/1.73m^2^) ^3^ | \ |
| **Living health variables** | smoking status | Yes (having smoked a minimum of 100 cigarettes throughout their life) |
|  | alcohol consumption | Yes (minimum of 12 alcoholic drinks annually) |
|  | hypertension | Yes (self-reported hypertension; use of antihypertensive medication; and mean systolic blood pressure ≥ 140 mmHg or mean diastolic blood pressure ≥ 90 mmHg.) |
|  | Hyperlipidemia | Yes (self-reported high cholesterol levels; use of cholesterol-lowering medications; and laboratory tests showing total cholesterol ≥ 200 mg/dl and HDL-C < 40 mg/dl (male) or < 50 mg/dl (female).) |
|  | diabetes | Yes (diabetes was defined as self-reported diabetes mellitus; insulin or glucose-lowering medications; hemoglobin A1c (HbA1c) ≥ 6. 5%; and fasting blood glucose (FBG) ≥ 7.0 mmol/L.)  Pre (prediabetes was defined as self-reported pre-diabetes mellitus; HbA1c 5.7-6.4%; and FBG 5.6-6.9 mmol/L.) |
|  | coronary heart disease (CHD) | Yes (self-reported as yes.) |
|  | myocardial infarction |  |
|  | stroke |  |
|  | chronic obstructive pulmonary disease  (COPD) |  |
|  | cancer |  |
|  | hypoglycemic therapy |  |
|  | cholesterol-lowering therapy |  |
|  | antihypertensive therapy |  |
