# Supplementary material for: Comparison of systemic immunoinflammatory biomarkers for assessing severe abdominal aortic calcification among US adults aged≥40 years: A cross-sectional analysis from NHANES
Source: PLoS One. 2025 Jun 24;20(6):e0325949. doi: 10.1371/journal.pone.0325949 (PMC12186907; doi:10.1371/journal.pone.0325949)
Supplement: S2 Table — (DOCX) [file pone.0325949.s002.docx]

**S2 Table** Kolmogorov-Smirnov tests for systemic immunoinflammatory biomarkers.

| **Exposure variables** | **D-statistic** | **P-value** | **Conclusion of normality** |
| --- | --- | --- | --- |
| SII | 0.140 | **<0.001** | Reject normality |
| SIRI | 0.163 | **<0.001** | Reject normality |
| AISI | 0.180 | **<0.001** | Reject normality |
| PLR | 0.142 | **<0.001** | Reject normality |
| NLR | 0.093 | **<0.001** | Reject normality |
| MLR | 0.141 | **<0.001** | Reject normality |
| Ln-SII | 0.024 | 0.056 | Not Reject normality |
| Ln-SIRI | 0.027 | **0.022** | Reject normality |
| Ln-AISI | 0.023 | 0.071 | Not reject normality |
| Ln-PLR | 0.035 | **0.001** | Reject normality |
| Ln-NLR | 0.024 | 0.059 | Not reject normality |
| Ln-MLR | 0.022 | 0.079 | Not Reject normality |
