# Supplementary material for: Comparison of systemic immunoinflammatory biomarkers for assessing severe abdominal aortic calcification among US adults aged≥40 years: A cross-sectional analysis from NHANES
Source: PLoS One. 2025 Jun 24;20(6):e0325949. doi: 10.1371/journal.pone.0325949 (PMC12186907; doi:10.1371/journal.pone.0325949)
Supplement: S3 Table — (DOCX) [file pone.0325949.s003.docx]

**S3** **Table** Multicollinearity assessment of covariates.

| **Variable** | **VIF value (before exclusion)** | **VIF value (after exclusion)** |
| --- | --- | --- |
| Age | 2.011668 | 1.921711 |
| Gender | 2.618242 | 2.472052 |
| Race/ethnicity | 1.185291 | 1.183237 |
| PIR | 1.360104 | 1.360103 |
| Education level | 1.422564 | 1.418962 |
| BMI | **5.429731** | 1.298286 |
| Waist circumference | **5.741693** | - |
| Grip strength | 2.525068 | 2.520766 |
| Total cholesterol | 1.456817 | 1.452991 |
| HDL-C | 1.472587 | 1.439340 |
| Vitamin D | 1.183658 | 1.183125 |
| eGFR | 1.303769 | 1.302928 |
| Smoking status | 1.201417 | 1.194442 |
| Alcohol consumption | 1.240953 | 1.239039 |
| Hypertension | 1.997313 | 1.995782 |
| Hyperlipidemia | 1.566820 | 1.566593 |
| Diabetes | 1.115272 | 1.115240 |
| CHD | 1.392684 | 1.391904 |
| Myocardial infarction | 1.351358 | 1.348841 |
| Stroke | 1.056787 | 1.056410 |
| COPD | 1.093939 | 1.085078 |
| Cancer | 1.111668 | 1.111612 |
| Hypoglycemic therapy | 1.211472 | 1.205449 |
| Cholesterol-lowering therapy | 1.740860 | 1.734889 |
| Antihypertensive therapy | 2.238445 | 2.238144 |
