# Supplementary material for: Comparison of systemic immunoinflammatory biomarkers for assessing severe abdominal aortic calcification among US adults aged≥40 years: A cross-sectional analysis from NHANES
Source: PLoS One. 2025 Jun 24;20(6):e0325949. doi: 10.1371/journal.pone.0325949 (PMC12186907; doi:10.1371/journal.pone.0325949)
Supplement: S4 Table — (DOCX) [file pone.0325949.s004.docx]

**S4** **Table** Subgroup and interaction analysis of the association between systemic immunoinflammatory biomarkers (SII, SIRI, AISI, PLR, NLR, MLR) and severe AAC.

| **Subgroup** | **lnSII** | | | **lnSIRI** | | | **lnAISI** | | | **lnPLR** | | | **lnNLR** | | | **lnMLR** | | |
| --- | --- | --- | --- | --- | --- | --- | --- | --- | --- | --- | --- | --- | --- | --- | --- | --- | --- | --- |
|  | **OR**  **(95% CI)** | ***P*** | **P-int** | **OR**  **(95% CI)** | ***P*** | **P-int** | **OR**  **(95% CI)** | ***P*** | **P-int** | **OR**  **(95% CI)** | ***P*** | **P-int** | **OR**  **(95% CI)** | ***P*** | **P-int** | **OR**  **(95% CI)** | ***P*** | **P-int** |
| **Age** |  |  | 0.778 |  |  | 0.857 |  |  | 0.877 |  |  | 0.475 |  |  | 0.413 |  |  | 0.542 |
| <60 years | 1.30 (0.71,2.37) | 0.392 |  | 1.39 (0.81,2.41) | 0.233 |  | 1.38 (0.86,2.21) | 0.177 |  | 1.22 (0.59,2.53) | 0.587 |  | 1.02 (0.44,2.33) | 0.967 |  | 1.34 (0.57,3.12) | 0.500 |  |
| ≥60 years | 1.19 (0.92,1.56) | 0.190 |  | 1.35 (1.05,1.75) | 0.021 |  | 1.22 (0.98,1.52) | 0.078 |  | 1.33 (0.98,1.80) | 0.070 |  | 1.15 (0.79,1.67) | 0.461 |  | 1.60 (1.09,2.36) | 0.018 |  |
| **Gender** |  |  | 0.674 |  |  | 0.210 |  |  | 0.638 |  |  | 0.675 |  |  | 0.291 |  |  | 0.188 |
| Male | 1.34 (0.93,1.92) | 0.116 |  | 1.34 (0.96,1.86) | 0.084 |  | 1.31 (0.97,1.75) | 0.075 |  | 1.32 (0.89,1.97) | 0.165 |  | 1.18 (0.71,1.95) | 0.530 |  | 1.32 (0.79,2.19) | 0.284 |  |
| Female | 1.05 (0.75,1.49) | 0.763 |  | 1.43 (1.02,2.00) | 0.039 |  | 1.20 (0.91,1.59) | 0.201 |  | 1.25 (0.82,1.89) | 0.300 |  | 0.94 (0.58,1.51) | 0.789 |  | 1.86 (1.12,3.11) | 0.017 |  |
| **BMI** |  |  | 0.571 |  |  | 0.704 |  |  | 0.381 |  |  | 0.952 |  |  | 0.816 |  |  | 0.820 |
| <25 kg/m2 | 1.01 (0.65,1.56) | 0.971 |  | 1.03 (0.68,1.57) | 0.878 |  | 0.95 (0.66,1.36) | 0.772 |  | 1.18 (0.71,1.95) | 0.530 |  | 1.15 (0.63,2.10) | 0.641 |  | 1.23 (0.66,2.29) | 0.510 |  |
| 25-30 kg/m2 | 1.32 (0.91,1.93) | 0.144 |  | 1.53 (1.06,2.20) | 0.023 |  | 1.39 (1.02,1.90) | 0.036 |  | 1.38 (0.90,2.13) | 0.144 |  | 1.12 (0.66,1.89) | 0.686 |  | 1.68 (0.96,2.96) | 0.070 |  |
| ≥30 kg/m2 | 1.40 (0.82,2.39) | 0.219 |  | 1.80 (1.10,2.95) | 0.019 |  | 1.58 (1.03,2.42) | 0.034 |  | 1.53 (0.81,2.89) | 0.188 |  | 1.16 (0.54,2.47) | 0.709 |  | 2.31 (1.10,4.85) | 0.027 |  |
| **Hypertension** |  |  | 0.374 |  |  | 0.694 |  |  | 0.345 |  |  | 0.919 |  |  | 0.842 |  |  | 0.761 |
| Yes | 1.13 (0.86,1.49) | 0.369 |  | 1.32 (1.03,1.71) | 0.030 |  | 1.20 (0.96,1.50) | 0.119 |  | 1.30 (0.94,1.79) | 0.112 |  | 1.08 (0.74,1.59) | 0.684 |  | 1.68 (1.13,2.50) | 0.011 |  |
| No | 1.39 (0.80,2.41) | 0.243 |  | 1.70 (0.94,3.05) | 0.079 |  | 1.41 (0.91,2.19) | 0.129 |  | 1.20 (0.64,2.25) | 0.57 |  | 1.03 (0.48,2.19) | 0.94 |  | 1.06 (0.48,2.36) | 0.882 |  |
| **Hyperlipidemia** |  |  | 0.544 |  |  | 0.706 |  |  | 0.499 |  |  | 0.841 |  |  | 0.083 |  |  | 0.246 |
| Yes | 1.16 (0.88,1.52) | 0.295 |  | 1.36 (1.04,1.77) | 0.023 |  | 1.20 (0.97,1.49) | 0.100 |  | 1.28 (0.93,1.75) | 0.127 |  | 1.30 (0.89,1.90) | 0.176 |  | 1.90 (1.28,2.81) | 0.001 |  |
| No | 1.31 (0.73,2.35) | 0.363 |  | 1.34 (0.81,2.24) | 0.257 |  | 1.48 (0.90,2.44) | 0.126 |  | 1.43 (0.72,2.85) | 0.312 |  | 0.48 (0.21,1.12) | 0.091 |  | 0.77 (0.31,1.92) | 0.578 |  |
| **Diabetes** |  |  | **0.026** |  |  | **0.033** |  |  | **0.036** |  |  | **0.035** |  |  | 0.190 |  |  | **0.032** |
| Yes | 1.84 (1.18,2.85) | 0.007 |  | 2.15 (1.43,3.23) | <.001 |  | 1.76 (1.23,2.50) | 0.002 |  | 2.21 (1.35,3.62) | 0.002 |  | 1.56 (0.86,2.82) | 0.145 |  | 2.75 (1.49,5.06) | 0.001 |  |
| No | 0.96 (0.60,1.55) | 0.880 |  | 1.20 (0.74,1.93) | 0.461 |  | 1.13 (0.75,1.69) | 0.566 |  | 0.97 (0.56,1.69) | 0.911 |  | 0.73 (0.36,1.46) | 0.372 |  | 1.16 (0.56,2.39) | 0.686 |  |
| Pre | 0.86 (0.57,1.30) | 0.469 |  | 0.96 (0.65,1.41) | 0.827 |  | 0.93 (0.66,1.30) | 0.661 |  | 0.89 (0.55,1.43) | 0.631 |  | 0.99 (0.57,1.71) | 0.971 |  | 1.20 (0.68,2.13) | 0.527 |  |
| **Cancer** |  |  | 0.454 |  |  | 0.619 |  |  | 0.352 |  |  | 0.846 |  |  | 0.918 |  |  | 0.930 |
| Yes | 0.93 (0.53,1.63) | 0.803 |  | 1.25 (0.74,2.11) | 0.403 |  | 1.04 (0.66,1.64) | 0.869 |  | 1.17 (0.63,2.16) | 0.628 |  | 1.11 (0.55,2.27) | 0.765 |  | 1.88 (0.92,3.84) | 0.083 |  |
| No | 1.28 (0.97,1.68) | 0.080 |  | 1.45 (1.11,1.88) | 0.006 |  | 1.34 (1.07,1.67) | 0.011 |  | 1.34 (0.97,1.85) | 0.072 |  | 1.13 (0.77,1.66) | 0.543 |  | 1.65 (1.09,2.48) | 0.017 |  |

OR: odds ratio.

95% CI: 95% confidence interval.

P-int: P for interaction.

Gender, age, race, PIR, education level, BMI, smoking status, alcohol consumption, grip strength, total cholesterol, HDL-C, Vitamin D, eGFR, hypertension, hyperlipidemia, diabetes, CHD, myocardial infarction, stroke, COPD, cancer, hypoglycemic therapy, cholesterol-lowering therapy, antihypertensive therapy were adjusted.
