# Supplementary material for: Comparison of systemic immunoinflammatory biomarkers for assessing severe abdominal aortic calcification among US adults aged≥40 years: A cross-sectional analysis from NHANES
Source: PLoS One. 2025 Jun 24;20(6):e0325949. doi: 10.1371/journal.pone.0325949 (PMC12186907; doi:10.1371/journal.pone.0325949)
Supplement: S5 Table — (DOCX) [file pone.0325949.s005.docx]

**S5** **Table** Spearman correlation of systemic immunoinflammatory biomarkers with abdominal aortic calcification of participants.

|  | **SII** | | **SIRI** | | **AISI** | | **PLR** | | **NLR** | | **MLR** | |
| --- | --- | --- | --- | --- | --- | --- | --- | --- | --- | --- | --- | --- |
|  | **R** | **P** | **R** | **P** | **R** | **P** | **R** | **P** | **R** | **P** | **R** | **P** |
| AAC score | 0.075 | <0.001 | 0.126 | <0.001 | 0.084 | <0.001 | 0.079 | <0.001 | -0.021 | 0.004 | 0.128 | <0.001 |
| Severe AAC | 0.106 | <0.001 | 0.153 | <0.001 | 0.111 | <0.001 | 0.120 | <0.001 | 0.023 | 0.002 | 0.154 | <0.001 |

Abbreviation: AAC, abdominal aortic calcification; SII, systemic immune-inflammation index; SIRI, system inflammation response index; AISI, aggregate index of systemic inflammation; PLR, platelet-to-lymphocyte ratio; NLR, neutrophil-to-lymphocyte ratio; MLR, Monocyte-to-lymphocyte ratio.
