# Supplementary material for: Comparison of systemic immunoinflammatory biomarkers for assessing severe abdominal aortic calcification among US adults aged≥40 years: A cross-sectional analysis from NHANES
Source: PLoS One. 2025 Jun 24;20(6):e0325949. doi: 10.1371/journal.pone.0325949 (PMC12186907; doi:10.1371/journal.pone.0325949)
Supplement: S6 Table — (DOCX) [file pone.0325949.s006.docx]

**S6** **Table** Diagnostic efficacy of systemic immunoinflammatory biomarkers (SII, SIRI, AISI, PLR, NLR, MLR) and constructed prediction model for severe AAC.

|  | AUC (95%CI) | Accuracy (95%CI) | Sensitivity (95%CI) | Specificity (95%CI) | PPV (95%CI) | NPV (95%CI) | Cut off |
| --- | --- | --- | --- | --- | --- | --- | --- |
| Model | 0.853 (0.832 - 0.874) | 0.770 (0.755 - 0.785) | 0.766 (0.750 - 0.782) | 0.809 (0.766 - 0.851) | 0.971 (0.963 - 0.978) | 0.295 (0.265 - 0.324) | 0.113 |
| MLR | 0.644 (0.631 - 0.657) | 0.718 (0.702 - 0.734) | 0.749 (0.732 - 0.765) | 0.465 (0.411 - 0.519) | 0.920 (0.909 - 0.932) | 0.183 (0.157 - 0.209) | 0.344 |
| SIRI | 0.643 (0.630 - 0.656) | 0.701 (0.684 - 0.717) | 0.724 (0.707 - 0.741) | 0.508 (0.454 - 0.562) | 0.924 (0.913 - 0.935) | 0.182 (0.157 - 0.207) | 1.442 |
| PLR | 0.611 (0.598 - 0.625) | 0.693 (0.676 - 0.709) | 0.720 (0.703 - 0.737) | 0.471 (0.417 - 0.525) | 0.918 (0.907 - 0.930) | 0.169 (0.145 - 0.193) | 2.442 |
| AISI | 0.603 (0.589 - 0.617) | 0.694 (0.677 - 0.710) | 0.720 (0.703 - 0.737) | 0.477 (0.423 - 0.531) | 0.919 (0.908 - 0.931) | 0.171 (0.147 - 0.195) | 338.200 |
| SII | 0.570 (0.556 - 0.584) | 0.728 (0.711 - 0.743) | 0.772 (0.756 - 0.787) | 0.365 (0.313 - 0.417) | 0.909 (0.898 - 0.921) | 0.162 (0.135 - 0.188) | 628.952 |
| NLR | 0.522 (0.507 - 0.536) | 0.617 (0.600 - 0.635) | 0.642 (0.624 - 0.660) | 0.416 (0.363 - 0.470) | 0.901 (0.888 - 0.914) | 0.123 (0.104 - 0.143) | 128.305 |
