# Supplementary material for: Comparison of systemic immunoinflammatory biomarkers for assessing severe abdominal aortic calcification among US adults aged≥40 years: A cross-sectional analysis from NHANES
Source: PLoS One. 2025 Jun 24;20(6):e0325949. doi: 10.1371/journal.pone.0325949 (PMC12186907; doi:10.1371/journal.pone.0325949)
Supplement: S7 Table — (DOCX) [file pone.0325949.s007.docx]

**S7** **Table** Lasso regression analysis results (best log(λ_1se_) = -4.157).

| **Characteristic** | **r** | **Characteristic** | **r** |
| --- | --- | --- | --- |
| Gender | - | COPD | - |
| Age | 0.0796 | Cancer | - |
| Race | 0.0853 | Hypertension | - |
| PIR | - | Hyperlipidemia | - |
| Education level | - | Diabetes | 0.1428 |
| BMI | 0.0182 | Antihypertensive therapy | 3.0629 |
| Alcohol consumption | - | Cholesterol-lowering therapy | 0.1716 |
| Smoking status | 0.2466 | Hypoglycemic therapy |  |
| Grip strength | - | Total cholesterol | - |
| CHD | 0.5926 | HDL-C | - |
| Myocardial infarction | - | Vitamin D | - |
| Stroke | - | eGFR | - |

Abbreviation: PIR, poverty income ratio; BMI, body mass index; CHD, coronary heart disease; COPD, chronic obstructive pulmonary disease; HDL-C, high-density lipoprotein cholesterol, eGFR, estimated glomerular filtration rate.
