# Supplementary figures and images for: Comparison of systemic immunoinflammatory biomarkers for assessing severe abdominal aortic calcification among US adults aged≥40 years: A cross-sectional analysis from NHANES
Source: PLoS One. 2025 Jun 24;20(6):e0325949. doi: 10.1371/journal.pone.0325949 (PMC12186907; doi:10.1371/journal.pone.0325949)

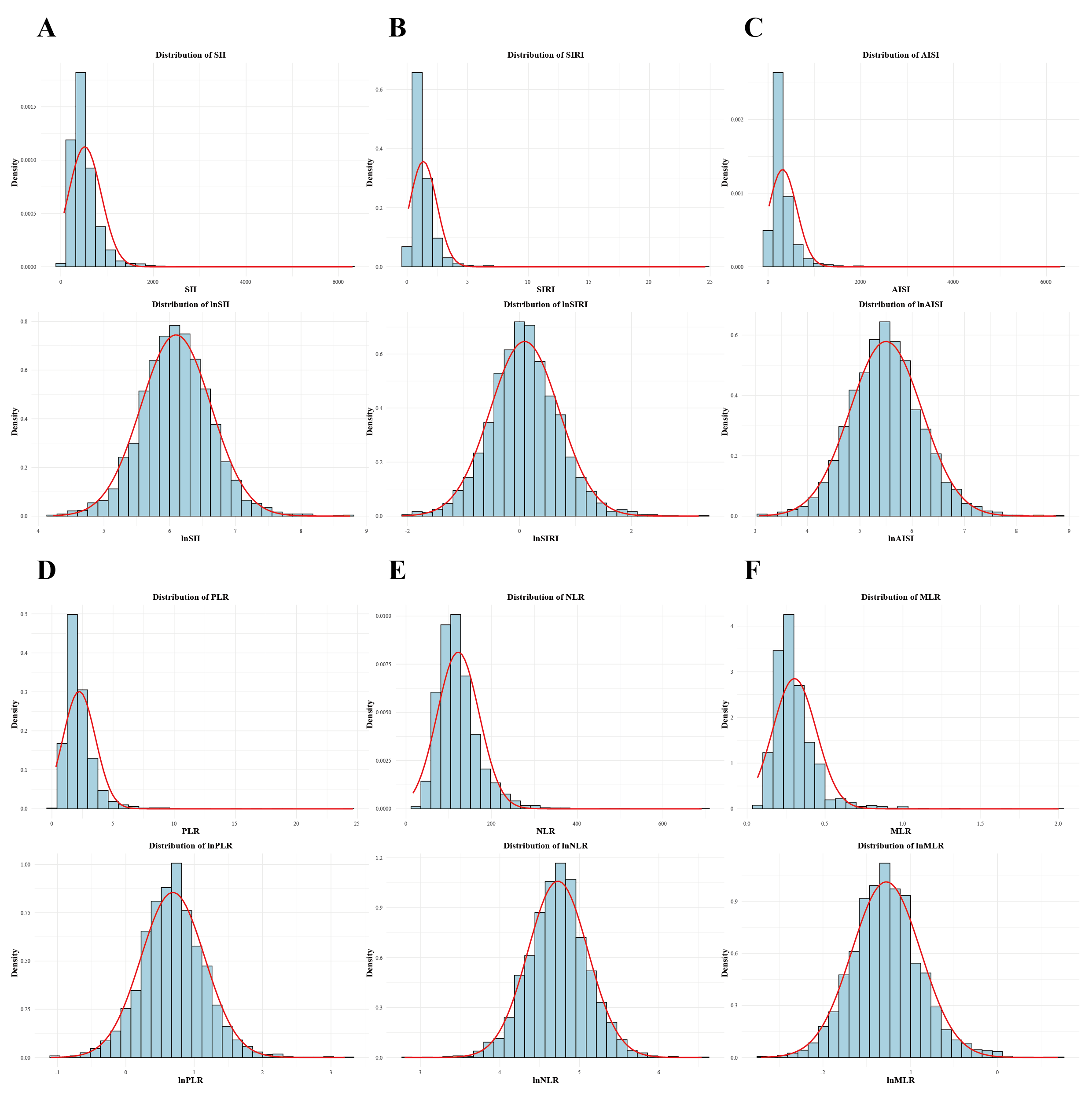

Supplement: S1 Fig — (A) SII and lnSII; (B) SIRI and lnSIRI; (C) AISI and lnAISI; (D) PLR and lnPLR; (E) NLR and lnNLR; (F) MLR and lnMLR. (TIF) [file pone.0325949.s008.tif]

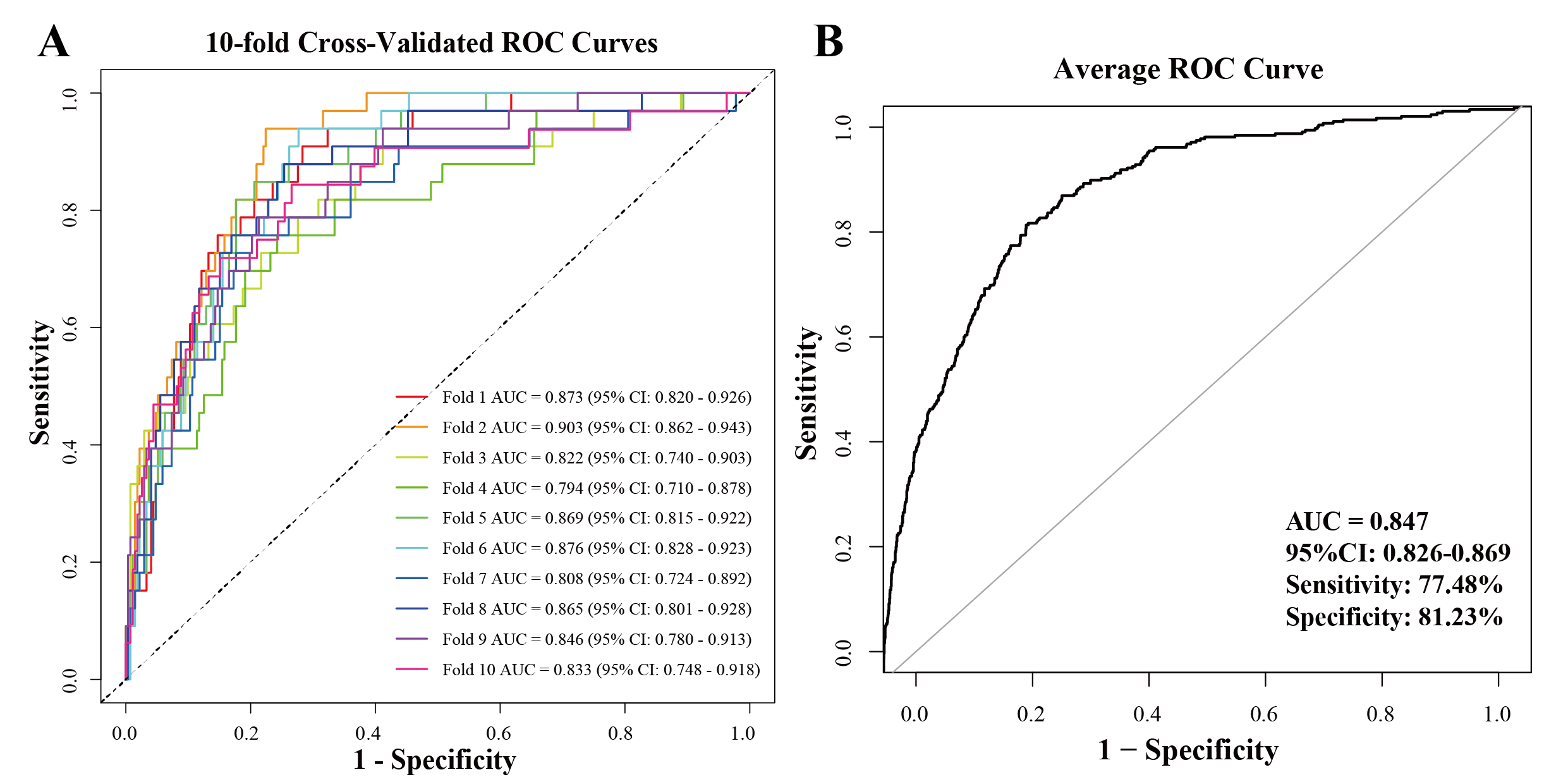

Supplement: S2 Fig — (A) ROC curve for each fold; (B) average ROC curve for all folds. (TIF) [file pone.0325949.s009.tif]
